# Pharmacological treatment with mirtazapine rescues cortical atrophy and respiratory deficits in MeCP2 null mice

**DOI:** 10.1038/srep19796

**Published:** 2016-01-25

**Authors:** Tamara Bittolo, Carlo Antonio Raminelli, Chiara Deiana, Gabriele Baj, Valentina Vaghi, Sara Ferrazzo, Annalisa Bernareggi, Enrico Tongiorgi

**Affiliations:** 1 Department of Life Sciences, University of Trieste, Via L. Giorgieri, 5-34127 Trieste, Italy

## Abstract

Loss of MeCP2 (Methyl CpG binding protein 2) in Rett syndrome (RTT) causes brain weight decrease, shrinkage of the cortex with reduced dendritic arborization, behavioral abnormalities, seizures and cardio-respiratory complications. The observed monoamine neurotransmitters reduction in RTT suggested antidepressants as a possible therapy. We treated MeCP2-null mice from postnatal-day 28 for two weeks with desipramine, already tested in RTT, or mirtazapine, an antidepressant with limited side-effects, known to promote GABA release. Mirtazapine was more effective than desipramine in restoring somatosensory cortex thickness by fully rescuing pyramidal neurons dendritic arborization and spine density. Functionally, mirtazapine treatment normalized heart rate, breath rate, anxiety levels, and eliminated the hopping behavior observed in MeCP2-null mice, leading to improved phenotypic score. These morphological and functional effects of mirtazapine were accompanied by reestablishment of the GABAergic and glutamatergic receptor activity recorded in cortex and brainstem tissues. Thus, mirtazapine can represent a new potential pharmacological treatment for the Rett syndrome.

Rett syndrome is an X-linked progressive neurodevelopmental disorder with an incidence of 1:10,000 newborn girls. Mutations in the gene encoding MeCP2 (Methyl CpG binding protein 2) are the main cause of the disorder^1^. The classical clinical course includes a normal development during the first two years of age and then a progressive general regression, with reduction of head growth, weight loss, repetitive stereotyped hand movements, motor disabilities, and cardiorespiratory dysfunctions representing the principal cause of death^2^.

Several clinical features are recapitulated in MeCP2^−/y^ (null) mice, including reduction in brain weight and volume^3^. A reduction in thickness of the somatosensory cortex was found in MeCP2-null mice at six weeks of age with shrinkage of layers II-III and V, accompanied by smaller neuronal size and reduced apical dendrite diameter^4^. Autistic-like behavior, motor and respiratory deficits were associated with a deficit of noradrenaline (NE), serotonin (5HT) and dopamine (DA) in Rett patients and MeCP2-null mice^5^,^6^, interconnected to a dysfunction of GABAergic and glutamatergic signaling^7^,^8^.

The alterations in monoaminergic signaling have suggested the possibility to ameliorate the Rett clinical phenotype through an antidepressant treatment. Desipramine, a tricyclic antidepressant inhibiting the reuptake of NE and 5HT, was shown to stabilize the respiratory pattern, and increase the life-span but without brain weight, body weight and locomotor activity improvement in MeCP2-null mice^9^. However, desipramine was reported to have severe side-effects, including cardiac complications^10^,^11^. On the other hand, mirtazapine, an high tolerable noradrenergic and specific serotoninergic tretacyclic antidepressant^12^, is virtually devoid of anticholinergic side-effects^13^, and has few cardiorespiratory side-effects although overdosage induces sedation^14^. Mirtazapine has been reported to enhance cognition^15^,^16^ and to stabilize sleep similarly to drugs that stimulate GABAergic transmission^17^. Here, MeCP2-null mice were treated with mirtazapine or desipramine for two weeks and the morphological and functional effects were analyzed.

## Results

### Reduction in body and brain weight and cortical thickness in MeCP2^−/y^ mice

**Figure 1:**
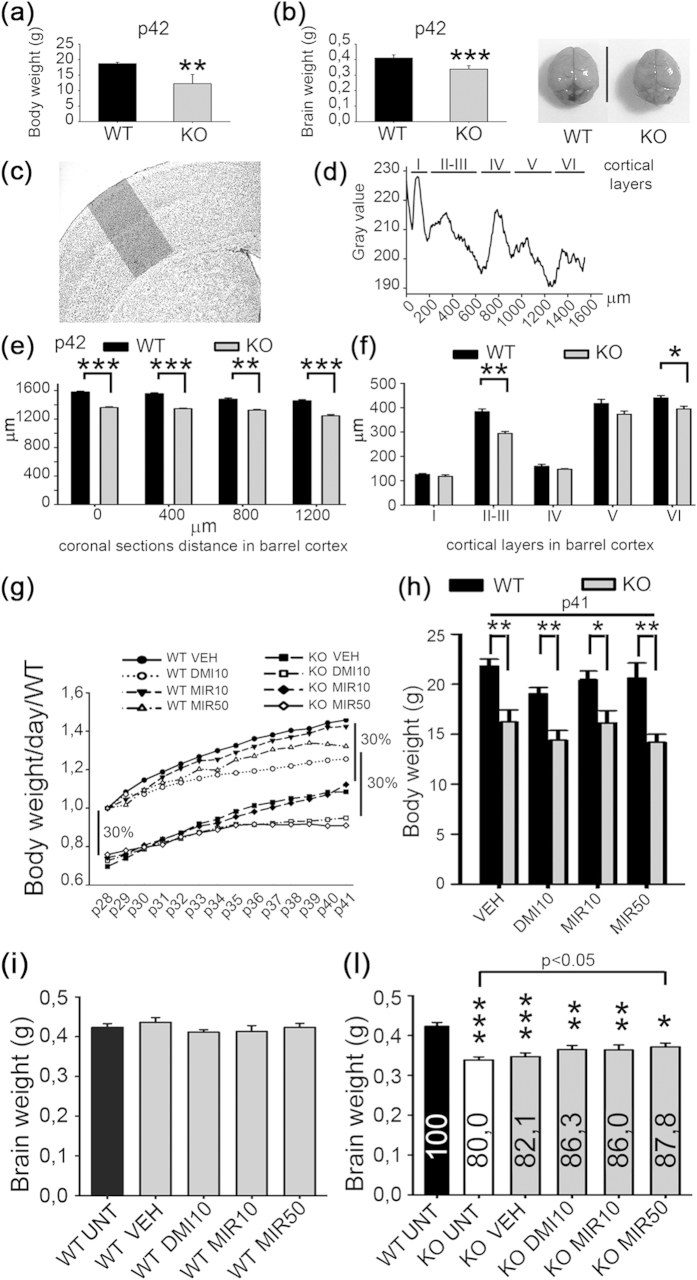
Body weight, brain weight, and thickness of somatosensory cortex in MeCP2^−/y^ mice . **(a,b)** Body and brain weight in grams of wild type (WT); MeCP2^−/y^ mice (KO), at p42 (n = 8–9). On the right, photographs of representative brains. Values are represented as mean ± SEM (**p = 0.002; *** p < 0.001; *t-test*). **(c)** Brain section stained with Nissl; the shadow indicate the area of somatosensory cortex used for densitometric analysis shown in **(d)**. Scale bar = 1000 μm. **(d)** Densitometric plot of gray values used to measure the total cortical thickness in μm and its individual layers, from I to VI. **(e)** Total cortical thickness (μm) in WT and KO mice from the beginning of the barrel cortex (0 μm) to 1200 μm (at intervals of 400 μm), at p42 (n = 6). Values are represented as mean ± SEM (**p = 0.002; ***p < 0.001; *t-test*). **(f)** Thickness of cortical layers (from I to VI) (μm) of WT and KO mice, at p42 (n = 6). Values are represented as mean ± SEM (*p = 0.022; ***p < 0.001; *t-test*). **(g)** Body weight of wild type (WT; n = 7) and MeCP2^−/y^ (KO; n = 7) mice treated with vehicle (VEH), desipramine 10 mg/Kg (DMI10 n = 8WT, 17KO) and mirtazapine 10 or 50 mg/Kg (MIR10 n = 6WT, 7KO; MIR50 n = 6WT, 15KO), measured every day from p28 to p41. Body weight of KO treated mice is normalized to the corresponding WT treated mice ( = 1 at p28). **(h)** Body weight evaluated at p41 in WT and KO treated mice. Values are represented as mean ± SEM; *p = 0.002 VEH; **p = 0.005 DMI10; *p = 0.017 MIR10; **p = 0.002 MIR50 (*t-test;* n = as in **(g)**). **(i)** WT brain weight of untreated (UNT) and treated mice with VEH, DMI10, MIR10 and MIR50 (n = 6–7; *One Way* ANOVA). **(l)** KO brain weight of untreated (WT UNT; KO UNT) and treated mice with VEH, DMI10, MIR10 and MIR50 (n = 5–8). Bars represent the mean ± SEM (*p < 0.05; *t-test*) of brain weight taking WT UNT as 100% (values in percentage are shown inside the bars).

MeCP2^−/y^ mice display body and brain loss of weight^18^. MeCP2^−/y^ male mouse model is generally considered a faithful model of the cortical dysfunction in Rett syndrome^19^. We used the Bird strain because it presents more significant alterations in brain weight, and volume of cortex and cerebellum, than those observed in the Jaenisch strain^3^. To obtain a reference before pharmacological treatment, we evaluated body and brain weight in MeCP2^−/y^ mice (KO) and Wild Type (WT) littermates at 6 weeks of age (p42). Compared to WT mice (n = 8), KO mice (n = 9) showed 34.7% reduction in body weight (WT = 18.73 ± 0.21 g; KO = 12.23 ± 1.05 g; *t-test* p = 0.002) and 19.04% reduction in brain weight (WT = 0.42 ± 0.01 g; KO = 0.34 ± 0.01 g; *t-test* p < 0.001) (Fig. 1a,b). Nissl stained brain sections (Fig. 1c,d; n = 6 WT; n = 6KO), collected at regular intervals every 400 μm from 0 μm until 1,200 μm along the antero-posterior axis of the somatosensory cortex, revealed significant reduction in the thickness of KO mice cortices (Fig. 1e, Suppl. Table 1; *t-test* p < 0.001 or p = 0.002 at interval 400–800 μm), and a significant reduction in layers II-III and VI compared with WT mice (Fig. 1f, Suppl. Table 1; *t-test*; layer II-III p < 0.001, layer VI p = 0.022) in agreement with previous studies^4^,^20^.

### Mirtazapine rescues the brain weight but not the body weight of MeCP2^−/y^ mice

Mirtazapine produces robust antidepressant effects already after two weeks of treatment^21^. To evaluate if antidepressants had an effect on body and brain weight, WT and MeCP2^−/y^ (KO) mice were treated for 2 weeks with vehicle (VEH), or the control drug (desipramine 10 mg/Kg – DMI10) or the testing drug at two different concentrations (mirtazapine 10 or 50 mg/Kg – MIR10/MIR50). Mice were weighed every day during the 14 days of treatment, and at p28, the KO-VEH group showed around 30% lower body weight with respect to the WT-VEH group and this difference was maintained until p41 irrespective of the drug treatment (Fig. 1g,h; n = 7 WT VEH = 21.81 ± 0.68 g; n = 8 WT DMI10 = 19.05 ± 0.62 g; n = 6 WT MIR10 = 20.46 ± 0.84 g; n = 6 WT MIR50 = 20.61 ± 1.50 g ; n = 7 KO VEH = 16.22 ± 1.20 g; n = 17 KO DMI10 = 14.40 ± 0.96 g; n = 7 KO MIR10 = 16.13 ± 1.22 g; n = 15 KO MIR50 = 15.20 ± 0.98 g; *t-test*, p = 0.002 VEH; p = 0.005 DMI10; p = 0.017 MIR10; p = 0.002 MIR50). Thus, the two drugs neither rescued nor reduced the body weight.

After 24 hours from the last injection, mice were euthanized and brains were weighed. The brain weight in untreated KO mice was 0.34 ± 0.008 g (n = 8 KO UNT), corresponding to 80% of the average brain weight in untreated WT mice (n = 6 WT UNT; 0.42 ± 0.01 g, t-test p < 0.001). The brain weight was not affected by drug treatments in WT mice (Fig. 1i; n = 6 WT UNT = 0.42 ± 0.01 g; n = 7 WT VEH = 0.44 ± 0.01 g; n = 7 WT DMI10 = 0.41 ± 0.01 g; n = 6 WT MIR10 = 0.41 ± 0.02 g; n = 6 WT MIR50 = 0.42 ± 0.01 g; One Way ANOVA, p = 0.503). Treatment of KO mice with vehicle had no significant improvement on brain weight with respect to KO UNT mice (n = 7 KO VEH = 0.35 ± 0.009 g, 82.1% of WT UNT; t-test). We observed a slight but not significant increase in brain weight with desipramine (DMI10 = 86.3%), and mirtazapine 10 mg/Kg (MIR10 = 86.0%) (n = 8 DMI10 0.36 ± 0.010 g, n = 6 MIR10 0.36 ± 0.013 g). However, treatment with mirtazapine 50 mg/Kg led to an increase in brain weight up to 87.8% of the weight in WT untreated mice, which was significantly different from KO UNT mice (Fig. 1l; n = 8 KO MIR50 0.37 ± 0.010; t-test p < 0.05) and was close to significance with respect to KO VEH (t-test p = 0.054). Thus, mirtazapine has a positive effect on the weight of RTT brains.

### Hippocampal structure is not affected by the loss of MeCP2 or drug treatments

To investigate which brain structures were involved in the increase of brain weight by mirtazapine treatment, we evaluated the effects of absence of MeCP2 and treatments on the hippocampus (Suppl. Fig. 1a). The specific contribution of each layer to the total thickness (=100%) is a constant along the rostro-caudal axis of the hippocampus in both mouse and rat, as previously demonstrated^22^. Using the same strategy, we analyzed three Nissl stained sections for each animal (n = 5 WT UNT, n = 5 KO UNT, n = 3 KO VEHIC, n = 3 KO DMI10, n = 3 KO MIR10, n = 4 KO MIR50) and measured the thickness of the different hippocampal laminas along a line drawn perpendicularly to the CA1 pyramidal layer (Suppl. Fig. 1b). The proportion of each layer in WT UNT resulted to be as follows, CA1 *stratum pyramidalis* = 8.7%, CA1 *stratum radiatum* = 32.5%, *stratum lacunosum-molecolare* = 19.6%, *stratum molecularis* = 27.3%, *stratum granularis* = 10.6%. No significant difference in thickness of the hippocampal layers was found between WT and KO mice or treatments (Suppl. Fig 1c, *One Way* ANOVA; p = 0.67 (pyr. l.); p = 0.56 (strat. ra); p = 0.91 (s. lac.); p = 0.95 (mol. l.); p = 0.37 (granul.)). Hence, the gross anatomical structure of the hippocampus was not affected by the loss of MeCP2 nor by treatment with antidepressants.

### Mirtazapine treatment rescues the thickness of somatosensory cortex in MeCP2^−/y^ mice

**Figure 2:**
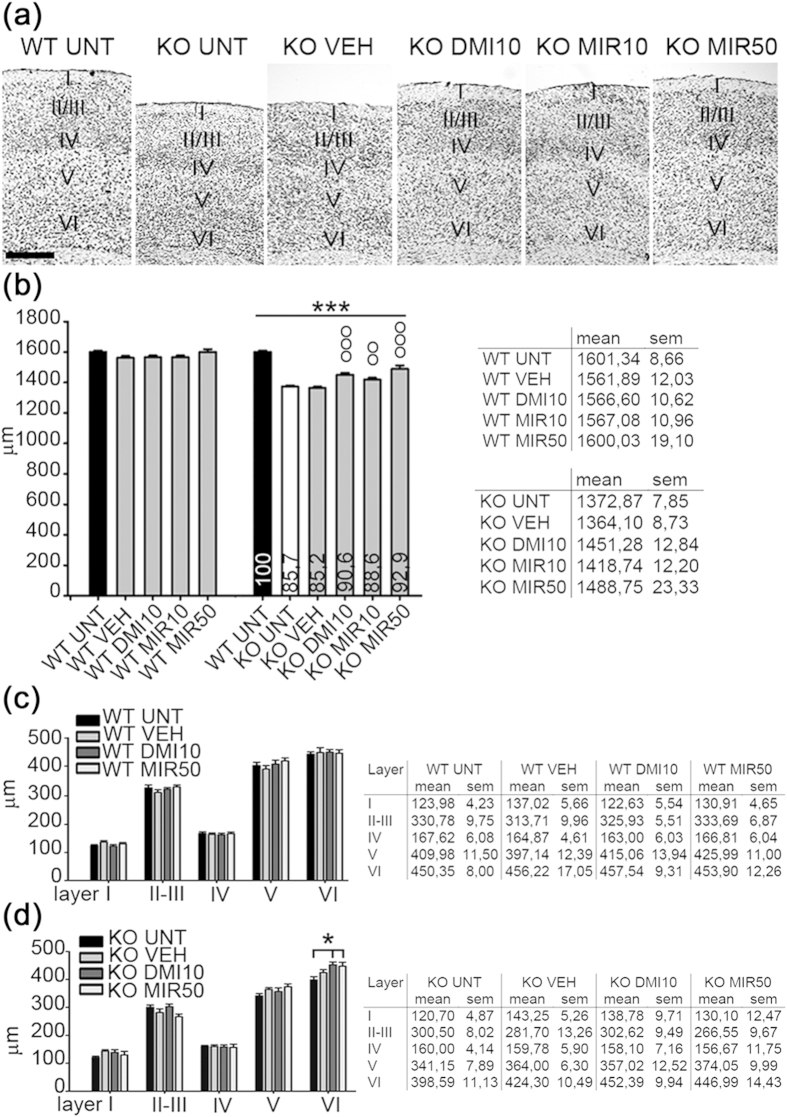
Total cortical thickness is rescued after the treatment with mirtazapine 50 mg/Kg. **(a)** Nissl staining of the somatosensory cortex in wild type untreated mice (WT UNT); MeCP2^−/y^ untreated mice (KO UNT); KO treated mice (VEH, DMI10, MIR10-50). Scale bar = 400 μm. **(b)** Total cortical thickness (μm) in WT and KO UNT mice and KO treated mice (n = 5–6). Bars are the mean ± SEM with respect to WT UNT mice ( = 100%; values in percentage are inside the bars). (*One way* ANOVA; °°°p < 0.001 KO DMI10; °°p = 0.038 MIR10; °°°p < 0.001 MIR50 vs. KO VEH; ***p < 0.001 vs. WT UNT). **(c,d)** Thickness of the layers in WT and KO mice untreated (UNT) or treated with VEH, DMI10 and MIR50 (μm) (n = 6). Values are represented in mean ± SEM and are shown in tables inserted in the figure (*One way* ANOVA; *p = 0.018 DMI10; *p = 0.021 MIR50 vs. KO UNT).

The thickness of somatosensory cortex was measured to determine if the pharmacological treatment could improve the abnormal cortical organization observed in MeCP2^−/y^ mice (Fig. 2a; n = 6 WT UNT; n = 6 WT VEH; n = 6 WT DMI10; n = 5 WT MIR10; n = 6 WT MIR50; n = 6 KO UNT; n = 6 KO VEH; n = 6 KO DMI10; n = 5 KO MIR10; n = 6 KO MIR50). In WT animals, mirtazapine and desipramine treatments showed no effects on cortical thickness (Fig. 2b; *One Way* ANOVA; p = 0.086). The total cortical thickness of KO untreated mice was 85.7% of WT (considered as 100%), and treated KO mice showed a statistically significant recovery of total cortical thickness compared with KO VEH (Fig. 2b; *One Way* ANOVA; KO DMI10, p = 0.001; KO MIR10, p = 0.038; KO MIR50, p < 0.001). The cortical thickness of KO mice treated with desipramine, mitarzapine 10 mg/Kg, or mirtazapine 50 mg/Kg reached the 90.6%, 88.6%, 92.9% of WT, respectively. When individual cortical layers were analyzed, treatments showed no effects in WT cortices (Fig. 2c, n = 6 for each group). However, we found an increase in the thickness of layer VI of somatosensory cortex in MeCP2^−/y^ mice treated with desipramine and mirtazapine 50 mg/Kg, compared with KO UNT (*One Way* ANOVA; KO DMI10 p = 0.018; KO MIR50 p = 0.021). These results confirmed that antidepressants treatment, in particular mirtazapine, can contribute to improve the cortical cytoarchitecture in MeCP2 null mice.

### Mirtazapine treatment rescues MeCP2^−/y^ cortical neurons morphology

**Figure 3:**
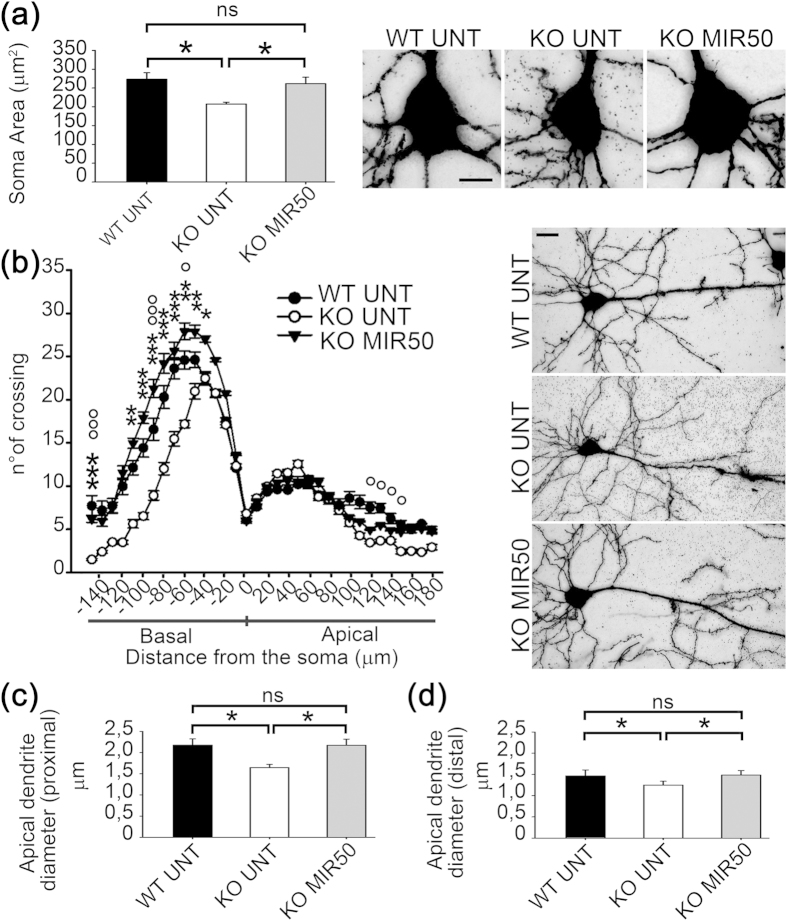
Golgi staining of layer II-III pyramidal neurons (somatosensory cortex) in WT and MeCP2^−/y^ untreated and treated mice. (**a**) Soma area of the neurons in wild type untreated mice (WT UNT); MeCP2^−/y^ untreated mice (KO UNT); KO MIR50-treated mice (n = 3 mice; n = 10 neurons for each mouse). Bars represent mean ± SEM (*p < 0.05; ns = not significant; ANOVA). On the right, examples of Golgi-stained neuronal somata, scale bar = 10 μm. **(b)** Sholl analysis of basal and apical dendritic crossing through a series of concentric circles centered at the soma and spaced at 10 μm intervals of WT/KO UNT and KO MIR50 mice is showed in the same graph. Values are represented as mean ± SEM. *KO MIR50 vs. KO UNT mice; °WT UNT vs. KO UNT mice; */°p < 0.05; **p < 0.01; ***/°°°p < 0.001 (ANOVA). On the right, examples of pyramidal neurons from WT UNT, KO UNT and KO MIR50 mice used for the Sholl analysis. Scale bar = 20 μm. **(c)** Apical dendrite diameter (proximal) at 10 μm from the soma in WT/KO UNT mice and KO MIR50 (*p < 0.05; ns = not significant; ANOVA). **(d)** Apical dendrite diameter (distal) at 100 μm from the soma in WT/KO UNT mice and KO MIR50 (*p < 0.05; ns = not significant; ANOVA).

To gain further insight regarding the effect of mirtazapine treatment on cortical neurons, we investigated the fine morphology of layer II-III pyramidal neurons of somatosensory cortex in MeCP2^−/y^ and WT mice using Golgi staining (Fig. 3, n = 10 neurons/animal, n = 3 mice/genotype; in total n = 30 neurons/genotype). Since treatments had no effect on WT cortices and thickness of KO cortex was not affected by the vehicle, in agreement with the 3 R principle for animal use reduction, we focused this analysis on WT untreated, KO untreated and KO treated with mirtazapine 50 mg/kg. In KO untreated mice, pyramidal neurons showed a significantly smaller area of their somata with respect to WT and this deficit was fully restored by mirtazapine 50 mg/Kg (WT UNT = 273.26 ± 17.54 μm^2^; KO UNT = 207.21 ± 5.08 μm^2^; KO MIR50 = 261.59 ± 17.35 μm^2^) (Fig. 3a; p < 0.05 ANOVA vs. KO untreated and not significant vs. WT UNT). By Sholl analysis, KO UNT mice showed a reduced number of crossings in the region from 120 to 150μm from the soma in apical dendrites (p < 0.05), and a marked reduction along the basal dendrites between −40 and −140 μm from the soma (p < 0.001; p < 0.05), with respect to WT UNT mice. Treatment with mirtazapine 50 mg/Kg rescued these deficits back to WT levels (Fig. 3b; p < 0.001, ANOVA).

In keeping with previous studies^4^, the diameter of apical dendrites of the layer II-III pyramidal neurons at 10 μm (proximal) and 100 μm (distal) from the soma showed a significant reduction in KO mice at both distance (Fig. 3c,d; p < 0.05 ANOVA). Treatment with mirtazapine led to a full recovery of this morphological deficit (Proximal: WT UNT = 2.18 ± 0.15 μm; KO UNT = 1.65 ± 0.08 μm; KO MIR50 = 2.17 ± 0.15 μm; Distal: WT UNT = 1.47 ± 0.14 μm; KO UNT = 1.25 ± 0.09 μm; KO MIR50 = 1.49 ± 0.11 μm) (Fig. 3c,d; p < 0.05, ANOVA), thus providing evidence that mirtazapine can restore the fine morphology of cortical neurons in MeCP2 null mice.

### Mirtazapine rescues the number and type of dendritic spines in MeCP2^−/y^

**Figure 4:**
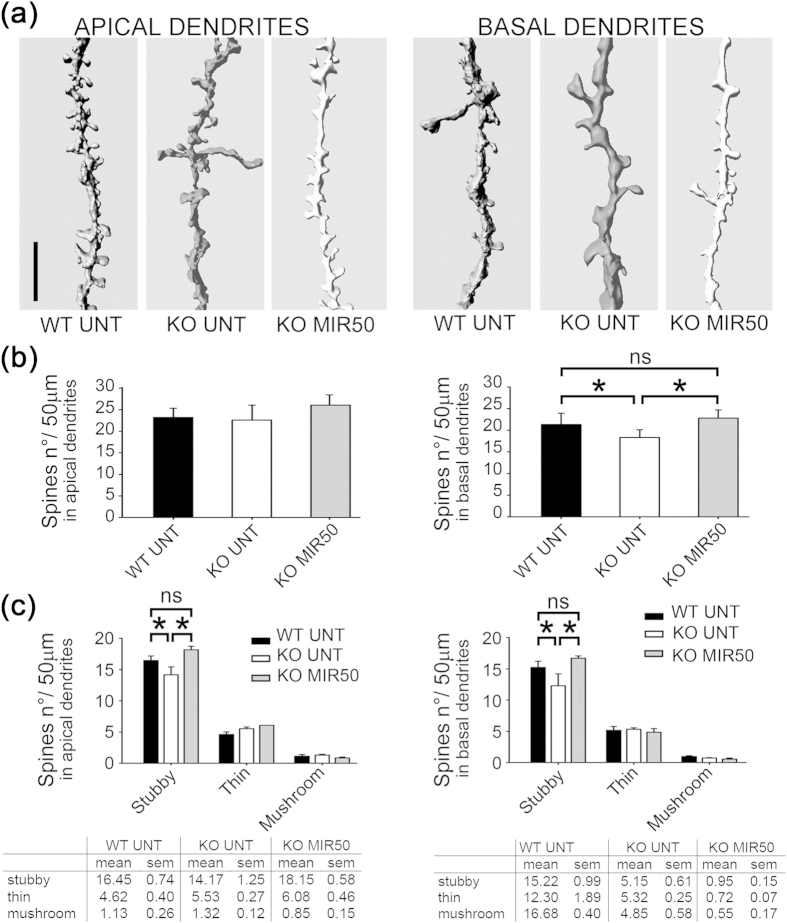
Mirtazapine rescues the number and type of dendritic spines in MeCP2^−/y^ neurons. **(a)** A 3D representation of secondary apical and basal dendrites of wild type untreated (WT UNT), MeCP2^−/y^ untreated (KO UNT) and KO mice treated with mirtazapine (MIR50). Scale bar = 5 μm. **(b)** Spine density (n° of spines in a dendrite segment of 50 μm) in apical and basal dendrites of WT/KO UNT mice and of KO MIR50 mice (n = 3 mice; n = 10 neurons for each mouse). Values are represented as mean ± SEM; *p < 0.05; ns = no significant (ANOVA). **(c)** Number of spines subdivided by spine type (stubby, mushroom and thin) in apical and basal dendrites (segment of 50 μm) of WT/KO UNT mice and KO MIR50 mice. Values are represented as mean ± SEM and are shown in the table at the bottom of the figure; *p < 0.05; ns = not significant (ANOVA).

It was previously reported that the density of spines in primary and secondary apical dendrites was similar between WT and KO mice (Jaenisch and Bird strains)^20^. Using sections labeled with Golgi staining, we investigated not only the spine density (number of spines/50 μm) in primary and secondary apical dendrites, confirming the results of previous studies (WT UNT n = 23.22 ± 2.12; KO UNT n = 22.60 ± 3.45), but also in basal dendrites wherein we observed a significant reduction in KO mice compared to WT (WT UNT n = 21.30 ± 2.61; KO UNT n = 18.32 ± 1.78) (Fig. 4a,b; n = 10 neurons/animal, n = 3 mice/genotype; in total n = 30 neurons/genotype). Treatment with mirtazapine 50 mg/Kg caused a complete rescue of basal dendrite spine density without altering the number of spines in apical dendrite (apical: KO MIR50 n = 26.07 ± 2.40; basal KO MIR50 n = 22.77 ± 1.90) (Fig. 4a,b; p < 0.05, ANOVA). When the spine type was considered, no difference in the number of mushroom and thin spines could be observed but the number of stubby spines resulted significantly reduced in both apical and basal dendrites from KO mice and was completely recovered by mirtazapine treatment (Fig. 4c; p < 0.05, ANOVA). In conclusion, mirtazapine treatment acts on the number and spine types to rescue the Rett phenotype on both apical and basal dendrites.

### Mirtazapine improves phenotypic score and cardiorespiratory functions

**Figure 5:**
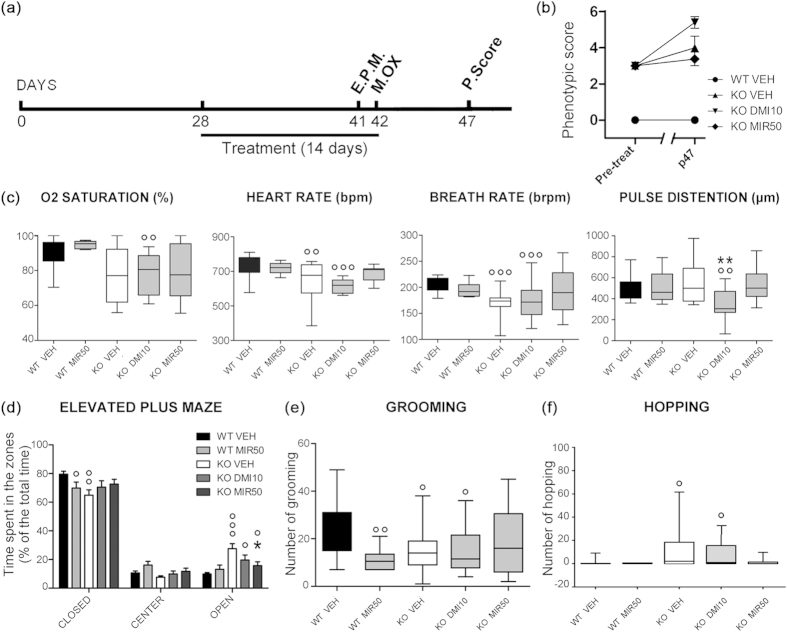
Assessment of functional outcomes of treatments. **(a)** Timeline for treatments and test battery. Elevated Plus Maze (E.P.M.) at p41, Mouse-Ox (M.OX.) at p42, Phenotypic score (P.Score) at p47. **(b)** Phenotypic score assessment pre- (p28) and post-treatment (p47) in WT and KO treated mice (n = 4–6) (*p < 0.05; *Mann-Whitney U test* 2-tailed). **(c)** Measurement of O2 saturation (in %), heath rate (bpm, beats per minute), breath rate (brpm, breaths per minute) and pulse distension (μm) calculated for 13–23 animals/group over an interval of 10 min (600 sec). (Statistical significance, *vs. WT VEH mice; ° vs. KO VEH mice; **/°°p < 0.01; °°°p < 0.001; *Mann-Whitney U test* 2-tailed). **(d)** Percentage of time spent in closed arms, center or open arms in the Elevated Plus Maze, for a test time of 10 min (n = 7–16 animals/group). **(e)** Number of grooming events and **(f)** hopping events over 20 min observation (n = 14–19 animals/group) (Statistical significance, °vs. WT VEH mice; *vs. KO VEH mice; */°p < 0.05; °°p < 0.01; °°°p < 0.001; *Mann-Whitney U test* 2-tailed).

Phenotypic outcomes of treatments were assessed starting from an evaluation of the general health conditions of animals at p28 and p47 (Fig. 5a). At p47, the group of mirtazapine-treated mice showed a lower phenotypic score (median = 3.5 ± 0.38; n = 4) in comparison to desipramine group (median = 4.5 ± 0.32; n = 5), indicating a significantly lower severity of disease symptoms (Fig. 5b; p < 0.05, *Mann-Whitney U test*). In the late stage of the disorder, RTT patients develop cardiorespiratory dysfunctions which are responsible for 26% of deaths^23^. To investigate if antidepressants treatments can modify the abnormal cardiorespiratory pattern of MeCP2-null mice, we used the MouseOX instrument^24^. The data continuously recorded during 10 minutes of the test are shown in Suppl. Fig. 2. Oxygen saturation in KO mice treated either with vehicle (median = 77.09 ± 0.3%; n = 14) or mirtazapine (median = 77.47 ± 0.9%; n = 15), was slightly, but not significantly, lower than WT injected with vehicle (median = 90.91 ± 0.3%; n = 18), while in KO treated with desipramine (median = 77.18 ± 0.5%; n = 15) it was significantly reduced (Fig. 5c; p < 0.01). On the contrary, in KO mice treated with vehicle, we observed a significant reduction in heart rate (beats per minute = bpm, median = 677.06 ± 2.4 bpm; n = 14; p < 0.01) and breath rate (breaths per minute = brpm, median = 173.61 ± 0.7 brpm; n = 14; p < 0.001) in comparison to WT mice treated with vehicle (median = 734.99 ± 1.3 bpm; median = 205.01 ± 0.9 brpm; n = 23). This deficit was unmodified by desipramine (median = 619.79 ± 6.2 bpm; median = 171.51 ± 0.7 brpm; n = 15; p < 0.001 vs. WT VEH) while it reverted back to WT levels after mirtazapine treatment (median = 710.08 ± 3.5 bpm; median = 196.24 ± 1.4 brpm; n = 13; not significant vs. WT VEH; Fig. 5c). We also recorded, for the first time in an animal model of Rett syndrome, the pulse distention. Pulse distention was not significantly altered in KO mice treated with vehicle (median = 500.62 ± 14.1 μm; n = 14) with respect to WT treated with vehicle (median = 434.41 ± 3.8 μm; n = 16), and mirtazapine (median = 501.38 ± 6.2 μm; n = 15) treatment did not affect this parameter. On the contrary, desipramine significantly reduced pulse distention in MeCP2^−/y^ mice (median = 304.97 ± 7.6 μm; n = 15; p < 0.01; Fig. 5c). Therefore, mirtazapine can rescue the irregular pattern of respiration described in both human and RTT mice, without eliciting unwanted side-effects.

### Mirtazapine normalizes anxiety and hopping behavior

MeCP2^−/y^ mice were previously shown to display reduced anxiety levels in the elevated plus maze^25^. Our results confirmed that KO mice treated with vehicle (KO VEH), spent more time in the open compartment of elevated plus maze (27.50 ± 3.56%; 82.51 ± 10.67 seconds; n = 14) than WT animals injected with vehicle (9.88 ± 0.98%; 29.64 ± 2.94 seconds; n = 16; p < 0.001) (Fig. 5d). KO mice treated with mirtazapine showed a significant reduction in the time spent in open arms (15.72 ± 2.67%; 47.17 ± 8.01 seconds; n = 13; p < 0.001) with respect to the KO VEH group, while desipramine was less effective (19.60 ± 3.54%; 58.79 ± 10.61 seconds; n = 13; p < 0.01). Mirtazapine did not produce any effect on WT animals (13.10 ± 3.03%; 39.29 ± 9.10 seconds; n = 7; Fig. 5d).

Another behavior which increases in response to elevated anxiety is self-grooming (Fig. 4e), which was significantly reduced in KO VEH mice (median = 14 ± 2.54 episodes; n = 15) with respect to WT VEH (median = 21 ± 2.57 episodes; n = 19; p < 0.01), in accordance with the increased preference for open arms observed in the elevated plus maze test. Treatment with mirtazapine 50 mg/Kg was able to partially restore the number of grooming episodes during observation (median = 16 ± 3.81 episodes; n = 14; p < 0.05 vs. KO VEH) while desipramine was not significantly different from the KO VEH group (median = 11.50 ± 2.91 episodes; n = 14; Fig. 5e).

Finally, we observed a typical behavior of MeCP2^−/y^ mice consisting in paroxysmal repetitive jump on the wall of the cage without control (hopping behavior), which is almost undetectable in WT VEH mice (1.44 ± 0.76 episodes; n = 18). Following treatment of KO mice with desipramine, the hopping behavior was less evident (7.57 ± 3.25 episodes; n = 14) than in KO VEH (12.5 ± 6.27 episodes; n = 14) while it was almost completely abolished after treatment with mirtazapine (1.54 ± 1.14 episodes; n = 14; Fig. 5f). The results regarding hopping behavior are particularly interesting because this uncontrolled jumping could be a sign of seizure events.

### Mirtazapine normalizes the GABA and glutamate-currents recorded in MeCP2^−/y^ cortical and brainstem tissues

To test if the reduction of hopping behavior, after the treatment with mirtazapine, could reflect a stabilization of cortical transmission, we investigated if mirtazapine could normalize excitatory and inhibitory neurotransmission in MeCP2^−/y^ mice. Transgenic mice lacking MeCP2 show reduced expression of GABAA receptor subunits^26^ and reduced GABA release in brain neurons^7^,^27^. Antidepressant treatments are known to rescue GABA levels and GABA deficits in patients with mood disorders^17^. Accordingly, the effects of mirtazapine on GABAA receptor functionality were investigated. After two weeks of treatment with mirtazapine (50 mg/Kg) or vehicle, MeCP2^−/y^ and WT mice were sacrificed and cell membranes were isolated from cortex and injected into Xenopus oocytes for electrophysiological analysis. This approach was chosen to measure the activity of all receptors expressed in the mouse cortex by using a limited number of animals. In this assay, the native receptors, still embedded in their natural lipidic environment with their associated molecules are transplanted into oocyte membranes maintaining their full functionality^28^,^29^. The oocytes were injected with the membranes isolated from the mouse cortices of n = 4 WT UNT, n = 3 KO UNT or n = 3 KO MIR50 p42 mice. Each sample was obtained from the cortex of a single mouse and injected into the oocytes at the same protein concentration. The currents were recorded from 1 to 4 days after injection. Following injection of the mouse brain membranes in oocytes, no treatment with mirtazapine was carried out.

**Figure 6:**
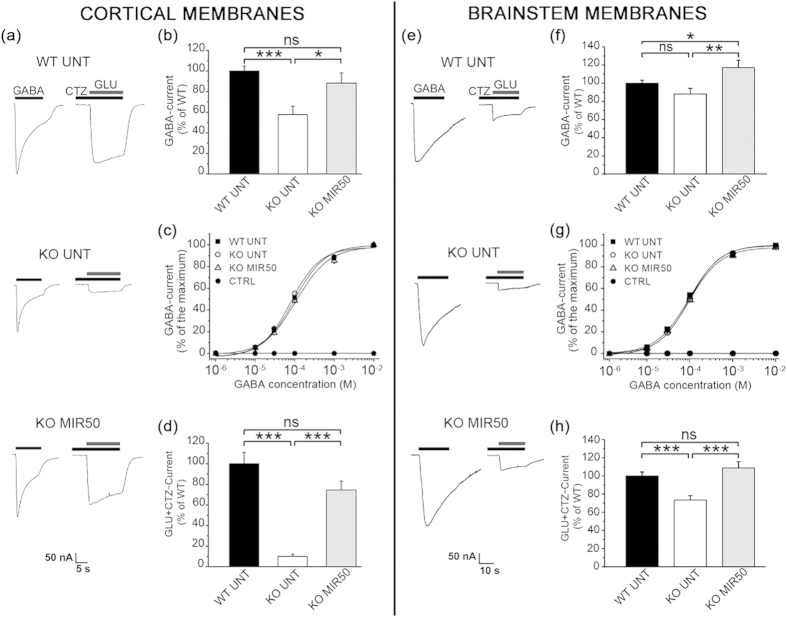
Mirtazapine treatment rescues GABA and GLU + CTZ currents in oocytes injected with membranes of MeCP2^−/y^ mouse cortex or brainstem. Representative traces of GABA(1 mM)- and GLU(1 mM) + CTZ(10 μM)-currents recorded in oocytes injected with cortical (**a**) or brainstem (e) membranes isolated from 3–4 WT UNT, 3 KO UNT and 3 KO MIR50 mice (CTZ was preapplied for 80 s to reduce glutamate receptor desensitization). Averages of GABA-current amplitudes (% of WT UNT) recorded in oocytes injected with cortical (**b**) or brainstem (**f**) membranes of WT UNT, KO UNT and KO MIR50 mice. GABA dose-current response relationships obtained from oocytes injected with cortical (**c**) or brainstem membranes (**g**), the values were normalized to the maximum value of inward currents (CTRL are values obtained from non-injected oocytes). (**d,h**) Averages of GLU + CTZ-currents (% of WT UNT) recorded in oocytes injected with cortex or brainstem membranes, respectively. Oocyte membrane potential was held at -80 mV. Values are represented as mean ± SEM; ns = not significant; *p < 0.05, **p < 0.01, ***p < 0.001 (*t-test*).

Figure 6a shows a typical example of GABA-current traces recorded in 3 oocytes, 2 days after injection with cortex membranes. In the same oocytes, glutamatergic-currents (GLU) were recorded in the presence of cyclothiazide (CTZ), a positive allosteric modulator that inhibits AMPA receptor desensitization^30^ often used to characterize the GLU receptors transplated into oocytes^29^,^31^. The currents were detected in all injected oocytes indicating the functional incorporation of the receptors. A lack of response was observed in non-injected cells (*data not shown*). As shown in Fig. 6b, the KO UNT-injected oocytes displayed a significant reduction of the GABA-current amplitude (WT UNT, 100 ± 4.77%, n = 80; KO UNT 57.67 ± 8.87%, n = 46, p < 0.001) whereas in KO MIR50-injected oocytes the amplitude was increased up to values comparable to those recorded in WT UNT-injected oocytes (88.24 ± 9.85%, n = 48). The dose-current response curves of Fig. 6c shows a left shift for KO UNT-injected oocytes revealing that the receptor affinity for GABA was altered. Half-maximal effective GABA concentration (EC_50_) of KO UNT-injected oocytes was significantly reduced compared to WT (WT UNT, EC_50_ = 98 ± 4.38 μM, *n*_H_ = 1.11 ± 0.04, n = 36; KO UNT, EC_50_ = 78.73 ± 4.92 μM, *n*_H_ = 1.15 ± 0.08, n = 39, p < 0.05). In KO MIR50-injected oocytes, the EC_50_ was rescued to WT levels (EC_50_ = 104.14 ± 11.2 μM, *n*_H_ = 1.02 ± 0.11, n = 14). The GLU + CTZ-current amplitude was significantly reduced in KO UNT-injected oocytes (Fig. 6d, WT UNT, 100 ± 12.73%, n = 12; KO UNT, 10.76 ± 1.87%, n = 12, p < 0.001), and was fully recovered in KO MIR50-injected cells (74.2 ± 8.93%, n = 12).

By using the same approach, GABA and GLU + CTZ currents were analyzed in brainstem cell membranes extracted from n = 3 WT UNT, n = 3 KO UNT and n = 3 KO MIR50 mice (Fig. 6e). GABA-currents recorded in KO UNT-oocytes showed a trend towards a decrease with respect to WT UNT-injected oocytes (Fig. 6f, WT UNT, 100 ± 3.51%, n = 90; KO UNT, 87.21 ± 5.93%, n = 77, p = 0.057), while EC_50_ and *n*_H_ (Fig. 6g) remained similar (WT UNT, EC_50_ = 93.74 ± 5.21 μM, *n*_H_ = 1.16 ± 0.05, n = 13; KO UNT, EC_50_ = 97.8 ± 4.30 μM, *n*_H_ = 1.24 ± 0.06, n = 18). In KO MIR50-injected cells, the currents were significantly increased (117.65 ± 81.68%, n = 79, p < 0.001) without changes in receptor affinity (EC_50_ = 95.10 ± 3.96 μM, *n*_H_ = 1.18 ± 0.07, n = 15) with respect to WT UNT and KO UNT-injected oocytes. The significant reduction in GLU + CTZ-currents recorded from KO-UNT injected oocytes (WT UNT, 100 ± 4.33%, n = 83; KO UNT, 73.47 ± 4.9%, n = 74, p < 0.001) was fully recovered in brainstem membranes isolated from mirtazapine-treated animals (108.75 ± 7.07%, n = 78, Fig. 6h).

## Discussion

At present, there is no cure for the Rett syndrome. Some approaches tried to reverse the symptoms of this disorder through reintroduction of MeCP2 gene or reactivation of the normal allele^32^,^33^. An alternative approach is through pharmacological treatments, and administration of neurotrophins or neurotransmitter precursors acting on factors downstream the MeCP2 function. A deficit in monoamine levels was reported in the brain and cerebrospinal fluid of both Rett patients and MeCP2-null mice^6^. Antidepressants are therefore considered as strong candidates for the Rett syndrome and this study provides significant evidence that the antidepressant mirtazapine has great potential for future clinical trials, as discussed below. Indeed, our findings demonstrate that 2 weeks of mirtazapine treatment is sufficient to increase brain weight and re-establish somatosensory cortex architecture, normalize cardio-respiratory patterns and anxiety levels, abolish hopping behavior and restore the glutamatergic and GABAergic transmission in both cortex and brainstem in p42 MeCP2-null mice.

Results presented here, showing an increased brain weight in KO mice treated with mirtazapine are in keeping with previous findings that patients chronically treated with antidepressants (including mirtazapine) show a significant increase in the volume of hippocampus^34^. In this context, the significant increase observed in the neuronal soma size and dendritic arborization in KO mice treated with mirtazapine may represent a possible mechanism contributing to the restoration of brain weight. In fact, measurement of cortical morphology represents a robust parameter to evaluate a possible pharmacological treatment since alterations in cortical morphology in MeCP2-null mice are highly reproducible in different laboratories, including ours, and closely resembles those observed in patients^19^. Indeed, simplified dendritic arbor structures of the layers II-III pyramidal neurons were found in the cortex of post-mortem brains from Rett patients^35^. Our results confirm previous findings that thickness of the somatosensory cortex of MeCP2-null mice, especially in layers II-III and V, and diameter of proximal and distal dendrites from soma are significantly reduced at p42 compared to WT mice^4^. We also confirmed the reduction in soma area of MeCP2-null mice neurons and the reduction in apical dendritic arborization with no changes in the total number of spines^20^. The fact that we observed a reduction of stubby spines in apical dendrites of KO mice, even if the spine density was not altered with respect to WT, could be explained by the variability of shapes assumed by the different spine types. This variability is highlighted when the mean values for different spine populations were compared among multiple samples, leading to a different significance with respect to the total number of spines^36^.

We analyzed also the arborization not only of apical dendrites, but also of basal dendrites which resulted to be the most affected in p42 MeCP2-null mice. We found that basal dendrites of MeCP2-null mice are less developed and have reduced number of stubby spines compared to WT mice.

Our experiments demonstrated that mirtazapine was able to restore the thickness of MeCP2-null mice somatosensory cortex, especially of layers II-III and VI. These macroscopic effects of mirtazapine were accounted by the full restoration of the normal microscopic structure of layer II-III pyramidal neurons including size of somata, diameter of apical dendrites, arborization of basal dendrites and density of spines in particular, of stubby spines.

We found that mirtazapine treatment in MeCP2-null mice was able to restore both the glutamatergic and GABAergic responses which were reduced in MeCP2-null cortical neurons, in keeping with previous studies^37^,^38^. The current reduction observed in oocytes injected with cortical membranes from MeCP2-null mice could be a consequence of many factors such as an impaired receptor-channel function or an altered receptor subunit composition. These results could be explained by an altered number of GABAergic synapses or by abnormalities in density and composition of GABAA receptors of the MeCP2-null mouse cortex^7^,^39^, given that the properties of the GABA and glutamate receptors in their native membrane are retained after transplantation into the oocytes^28^,^29^. Therefore, our results should be considered in line with the reported reduction in GABAA receptor density in young human brains affected by Rett Syndrome and in the cerebrum of MeCP2 deficient mouse^26^,^40^,^41^. Such change could be also related to the different GABA affinity of the GABA receptors that we found in this study, in analogy to what observed in cortical tissue deriving from Angelmann syndrome patients^42^. In addition, we showed that mirtazapine was able to normalize GLU-currents, in agreement with the reported reduction in glutamate transmission in the cortex of MeCP2-null mice^8^.

Deficits in the monoamine system, involving norepinephrine (NE) and serotonin (5-HT), in brainstem are linked to breathing disturbances and NE application on brainstem slices of MeCP2-null mice is able to restore a regular respiratory rhythm^43^. Moreover, desipramine is able to rescue respiratory rhythm and extend the life-span in a mouse model of the Rett syndrome^9^. Mirtazapine is a noradrenergic and specific serotoninergic antidepressant able to increase BDNF levels^44^ and to induce the release of serotonin through the modulation of α2-adrenoreceptors^45^, thus modulating the cardiorespiratory system. In the present study, we found that mice treated with mirtazapine showed a better phenotypic score than those treated with desipramine or untreated. In addition, mirtazapine was able to rescue both the heart and breath rate deficits observed in MeCP2-null mice, without affecting O_2_ saturation and pulse distention which, together with the heart rate, are significanlty reduced by desipramine. Therefore, mirtazapine can rescue the irregular pattern of respiration, described in both human and RTT mice, without eliciting unwanted cardiovascular side-effects. Desipramine was shown to reduce apneas in MeCP2-null mice and to prolong their survival^9^. Nevertheless, desipramine may induce autonomic dysfunction leading to inability to control blood pressure, prolonged QT intervals, heart failure, and sudden cardiac death in predisposed children and adolescents^10^. In particular, prolonged QT intervals are caused by the ability of desipramine to bind to the hERG K + channel in hearth muscles^46^. This is a worrying side-effect considering that cardiorespiratory dysfunction with prolongation of the QT interval is the main cause of death of RTT patients^23^. Therefore, an alternative drug is highly needed and mirtazapine appears as a strong candidate because of its limited respiratory and cardiac effects including very little orthostatic hypotension^47^.

Our recordings of whole brainstem membranes showed significantly decreased glutamatergic responses, and a slight reduction of GABAergic transmission, and these deficits were completely rescued in whole brainstem membrane preparations from mirtazapine-treated animals. Previous studies reported an imbalance between inhibitory and excitatory synaptic transmission in the ventrolateral medulla at postnatal day 7 likely due to decreased GABA release and GABAA receptors^48^. In addition, treatments which enhanced GABAergic transmission improved respiratory patterns^49^ alike the effect observed with a BDNF-mimic molecule^50^. Although we cannot compare our results with these previous studies because our whole brainstem preparations do not allow for resolution of neurotransmission activity in specific neuronal populations, the functional effects on breathing and heath-beat control, together with a clear normalization of both GABAergic and glutamatergic currents in the brainstem, represent a strong evidence of the efficacy of mirtazapine treatment.

Although normally, the antidepressant effects are visible after few hours from the treatment, several studies demonstrated that chronic treatment (>14 days) with mirtazapine induces the increase of neurological responsiveness^44^,^51^. Besides, Lee *et al*., 2013 described that SSRI and tricyclic antidepressant show an evident therapeutical effect with a chronic (14 or more days) but not acute (<10 days) treatment^52^. To evaluate the behavioural effects of chronic treatment with mirtazapine, we decided to test MeCP2-null mice when the symptoms of the pathology are evident, that is from the 5–6th weeks of age^18^.

Mirtazapine treatment was shown to display a nootropic effect in schizophrenic patients^16^ due to increased release of NA and DA in prefrontal cortex^53^. In this context, it is interesting to note that mirtazapine also normalized the anxiety behavior. MeCP2 mutant mice were described to spend increased times in open arms of the elevated plus maze, an abnormal behavior which has been explained as due to abnormal perception of safeness or incapability to interpret danger in the MeCP2-null mice, two functions which require higher cognitive functions^54^. Similar results on anxiety and Glutamate normalization were obtained by treating MeCP2-null mice with IGF1^24^. One possible explanation of these similarities as well as those observed for breathing and the recovery of brainstem hyperexcitability could be that mirtazapine may act through mobilization of one or more neurotrophic factors, an hypothesis which is worth of further investigations. In conclusion, our results strongly support mirtazapine as a candidate drug to treat the Rett syndrome.

## Materials and Methods

### Mice

Animals were treated according to the institutional guidelines in compliance with the European Council Directive 86/609 and NIH Guide for the Care and Use of Laboratory Animals. Animal use was approved by the Italian Ministry of Health with authorization n°218/2012-B specific for this study, in compliance with the Italian law D.Lgs.116/92. Wild-type (WT) C57/BL6 male mice (Charles River Laboratories, Calco, LC, Italy) were crossed with female MeCP2 heterozygous mice (Jackson Laboratories, Bar Harbor, Maine; strain: B6.129P2(C)-Mecp2tm1.1Bird/J, stock: 003890) to obtain the Rett model mice with genotype MeCP2^−/y^^18^. We used male hemizygous mice because they consistently display the most evident and severe symptoms of the disorder much earlier than heterozygous female mice^19^. MeCP2 is an X-linked gene, and thus, heterozygous female mice show a different MeCP2 expression depending on the mosaicism derived from the X-chromosome inactivation (XCI) pattern^55^. Even if, it is likely that the hormonal changes could influence the outcome of the pathology, the variability of the phenotype due to the XCI in female mice could be confounding. All mice were kept in ventilated caging under 12:12 h light/dark cycle with food and water ad libitum. To improve pups survival rate, we used a cross-fostering approach in which mice offspring were raised by FVB foster mothers (Harlan Laboratories, Udine, Italy).

### B6.129P2(C)-Mecp2tm1.1Bird/J mice genotyping

Genotypes were assessed by PCR on tail genomic DNA using specific primers (forward common primer oIMR1436 5′- GGT AAA GAC CCA TGT GAC CC -3′, reverse mutant primer oIMR1437 5′- TCC ACC TAG CCT GCC TGT AC -3′, reverse wild type primer oIMR1438 5′- GGC TTG CCA CAT GAC AA -3′). PCR reactions were performed with 1U GoTaq Polymerase (Promega Corporation, Madison, WI, USA), 1X Green GoTaq Buffer, 0,2 mM dNTPs each, 2,5 mM MgCl_2_, 0,5 μM of each primer and 100 ng of genomic DNA, as follows: 5′ 95 °C, 30 cycles 45″ 95 °C, 50″ 57,5 °C, 50″ 72 °C, followed by 10′ 72 °C. The PCR generates a 400-bp product for WT, and 400 and 416-bp products for heterozygous mice, and a 416-bp product for hemizygous mice.

### Animal treatment

Beginning from p28, WT males and MeCP2^−/y^ (KO) littermates were treated daily with an i.p. injection for 14 days, at 10–11 a.m., with Vehicle (VEH = 1% aqueous solution of Tween80, Sigma-Aldrich, St. Louis, MO, USA), desipramine 10 mg/Kg (DMI10; Vinci-Biochem, Florence, Italy), mirtazapine 10–50 mg/Kg (MIR10–50, Abcam, Cambridge, UK). Each group was randomized on the basis of weight and the analyses were carried out by experimenters blinded for treatments according to the recommended guidelines for proper pre-clinical testing^19^,^56^.

### Nissl staining

Brains frozen in isopentan (n = 3–6) were cut in 20 μm coronal sections from the somatosensory cortex (S1-M1,barrel cortex; 1,32 mm to −1,64 mm from bregma) with a cryostat (Leica, Solms, Germany) and mounted in gelatin-coated microscope slides and left to dry. Sections were post-fixed in PBS/PFA 4% (30 minutes; 4 °C), washed 3 times in PBS and pre-incubated in PBS with 0,2% gelatin, 0,2% Tween20 (30 minutes). Nissl staining (0,2% cresyl violet, 0,5% acetic acid, 0,01 M sodium acetate in sterile water) was performed for 20 minutes at 37 °C. Sections were dehydrated in ethanol 70%, 95%, 100%, methanol, methanol-xylene (50:50), xylene and mounted with Eukitt (Sigma-Aldrich).

### Morphometric measurements

Thickness of the total cortex and of its layer of WT and KO mice untreated or treated with antidepressants were analyzed on 3 adjacent sections, taken from the beginning of the barrel cortex to 1200 μm. Pictures were taken using a high-definition Nikon AMX1200 digital camera on a Nikon E800 Microscope (10X magnification). Gray level profile along a line (width = 300) spanning perpendicularly the barrel cortex or the hippocampus was determined by ImageJ software.

### Golgi staining

We used a modification of the protocol by Ranjan and Mallick^57^. Brains (n = 3 mice/genotype) immersed in the Golgi solution (5% of Potassium Dichromate, 5% of Mercuric Chloride, 5% of Potassium Chromate) in the dark for 27 hours at 37 °C, were cut with a vibratome (Campden Instruments, Loughborough, UK; MA752 motorized advance vibroslice) in 200 μm coronal sections. A Z-series of images from 10 neurons (each mice) were collected every 1 μm at 60X magnification and analyzed using ImageJ software. The soma area was analyzed as a polygonal selection of the soma including the hillock. The diameter of the apical dendrite was measured at 10 or 100 microns from the soma. For each neuron, the number of thin, stubby and mushroom spines^58^ were evaluated in 2 segments of 50 μm of secondary basal dendrites or primary and secondary apical dendrites.

### Sholl analysis

Images of neurons (n = 10/mouse; n = 3 mice/genotype) were collected at 20X magnification with Nikon E800 microscope. A bidimensional image composed a series of stacks every 2 μm was analyzed with Neurostudio program (Computational Neurobiology and Imaging Center (CNIC). The Sholl analysis was performed separately for the basal and apical dendrites with concentric circles of 10 μm.

### Electrophysiological recordings from Xenopus oocytes

Cell membranes from p42 WT (n = 4), KO untreated (n = 3) and KO MIR50-treated (n = 3) mice cortices and brainstem were isolated and injected into Xenopus oocytes as previously described^59^. Xenopus laevis animal care and treatment were conducted in conformity with institutional guidelines in compliance with national and international laws and policies (European Economic Community (EEC) Council Directive 63/2010.italian D.L.26/2014). After defolliculation with collagenase (0,5 mg/ml, 35 minutes, Type I, Sigma-Aldrich) the oocytes were maintained at 16 °C in Barth’s solution containing 0,5 mg/ml gentamicin (Sigma-Aldrich) over-night. Membrane preparations were injected into the cells at the same protein concentration (1 mg/ml = 50 nl). One to four days after injection, the currents were recorded from voltage-clamped oocytes, using two microelectrodes filled with 3 M KCl^60^. During recordings the oocytes were continuously superfused at room temperature with Ringer solution (115 mM NaCl, 2 mM KCl, 1,8 mM CaCl_2_, 5 mM Hepes, adjusted to pH7 with NaOH) in a purpose-designed recording chamber (RC-3Z, Warner Instruments, Hamden, CT, USA). Data acquisition and analyses were performed using WinWCP version 3.5 Strathclyde Electrophysiology software (kindly provided by John Dempster, Glasgow, UK). Ligands (GABA = gamma-aminobutiric acid or GLU + CTZ = glutamic acid + cyclothiazide) were applied using a constant perfusion system (5–10 ml/min, VC-8 perfusion system, Warner Instruments). To reduce the variability, the current amplitudes recorded in oocytes injected with KO untreated or KO MIR50-treated membranes were normalized to that recorded in oocytes injected with WT untreated membranes of the same batch and at the same post injection day.

For dose/current-response curves, the GABA was repeatedly applied at 5 minutes intervals, and the half-dissociation constants (EC_50_) and Hill coefficients (*n*H) were estimated by fitting the data to Hill equations.

### Phenotypic scoring

Mice were removed from their home cages and placed onto a laboratory bench for observation at the same time of the day. Phenotype severity was evaluated through a score system previously described^33^. The mobility, the gait, the hind limb clasping, the tremor, the breathing and general condition were observed in WT and KO mice before drugs treatment, and again at p47. Scoring was as follow: 0 = absent or as WT; 1 = moderate phenotype (symptom present); 2 = severe phenotype (symptom severe). The resulted phenotype was calculated by the sum of the scores of each mouse and then averaging all animals within each group.

### Elevated Plus Maze Test

The apparatus was set as previously described^25^. Postnatal-day 41–42 mice (n = 7–16) were tested for 5 minutes (10–13 a.m.). Each test the cross was clean with ethanol 70% to avoid influencing the performance among mice. Data were analyzed with Any-maze program (Ugo Basile instrument, Varese, Italy) dividing the cross into central zone, open arms, closed arms. The percentage of the time spent in the zones vs the total time of the test, the number of grooming and the frequency of hopping behaviour were analyzed.

### Vital parameters measurements

The Oxygen Saturation (% = percentage of hemoglobin saturated by O_2_), the Heart (bpm = number of beats per minute) and Breath Rate (brpm = number of breaths per minute) and the Pulse Distention (μm = distention of the arterial blood vessels due to a cardiac pulse) were detected by the non-invasive MouseOX instrument (STARR Life Sciences, Holliston, Ma, USA). A collar clip was placed on the neck of p42 mice (n = 5–22) keeping for 20 minutes, and then substitued with the infrared pulse oximeter clip connected to the instrument. Ten minute readings were taken from each mouse. We excluded from the analysis the data equal to zero and the animals with O_2_ saturation lower than 50%, because these data are due to signal failure in the infrared signal.

### Statistical analysis

Statistical analyses were conducted using SigmaPlotTM software (SigmaStat, Systat Software Inc, San Jose, CA, USA).

The Student’s t-test was used for two-sample comparisons. For multiple sample comparisons, *One-Way* ANOVA was performed, followed by Student-Newman-Keuls Method post-tests. ANOVA on Ranks was used for morphological analysis of Golgi staining.

For behavioral analysis, outlier exclusion was performed through GraphPad Software (GraphPad Software Inc., La Jolla, CA, USA) with a Grubbs’ test, considering critical value of Z higher than 2.5. The non-parametric data obtained with MouseOX and behavioral test were analyzed with Mann-Whitney U test (2-tailed) between each group and the control.

## Additional Information

**How to cite this article**: Bittolo, T. *et al*. Pharmacological treatment with mirtazapine rescues cortical atrophy and respiratory deficits in MeCP2 null mice. *Sci. Rep.* **6**, 19796; doi: 10.1038/srep19796 (2016).

## Supplementary Material

Supplementary Information
