# Supplementary material for: Pharmacological treatment with mirtazapine rescues cortical atrophy and respiratory deficits in MeCP2 null mice
Source: Sci Rep. 2016 Jan 25;6:19796. doi: 10.1038/srep19796 (PMC4726391; doi:10.1038/srep19796)
Supplement: Supplementary Information [file srep19796-s1.pdf]

Suppl. Table 1.

Cortical interval	WT UNT	KO UNT	t-test
0-200 µm	1581±11.3 µm	1362±12 µm	p<0.001
200-400 µm	1557±12.5 µm	1346±9.03 µm	p<0.001
400-800 µm	1480±17.4 µm	1325±11.4 µm	p=0.002
800-1200 µm	1458±14.8 µm	1246±17.7 µm	p<0.001
**Cortical layer**	**WT UNT**	**KO UNT**	**t-test**
layer I	125.57±4.45 µm	117.8±6.51 µm	N.S.
layer II-III	383.68±11.12 µm	294.27 ± 7.98 µm	p<0.001
layer IV	159.65±7.8 µm	147.3 ± 2.4 µm	N.S.
layer V	416.98±17.33 µm	372.94 ± 13.01 µm	N.S.
layer VI	440.18±9.54 µm	395 ± 11.25 µm	p=0.022

Somatosensory cortical thickness in Wild Type and MeCP2 null untreated mice.
Measurements of total cortical thickness (in µm) at regular intervals every 400 µm 0 µm until 1,200 µm along the antero-posterior axis and measurements of cortical layers (from I to VI). Statistical analysis: t-test.

Suppl. Figure 1.

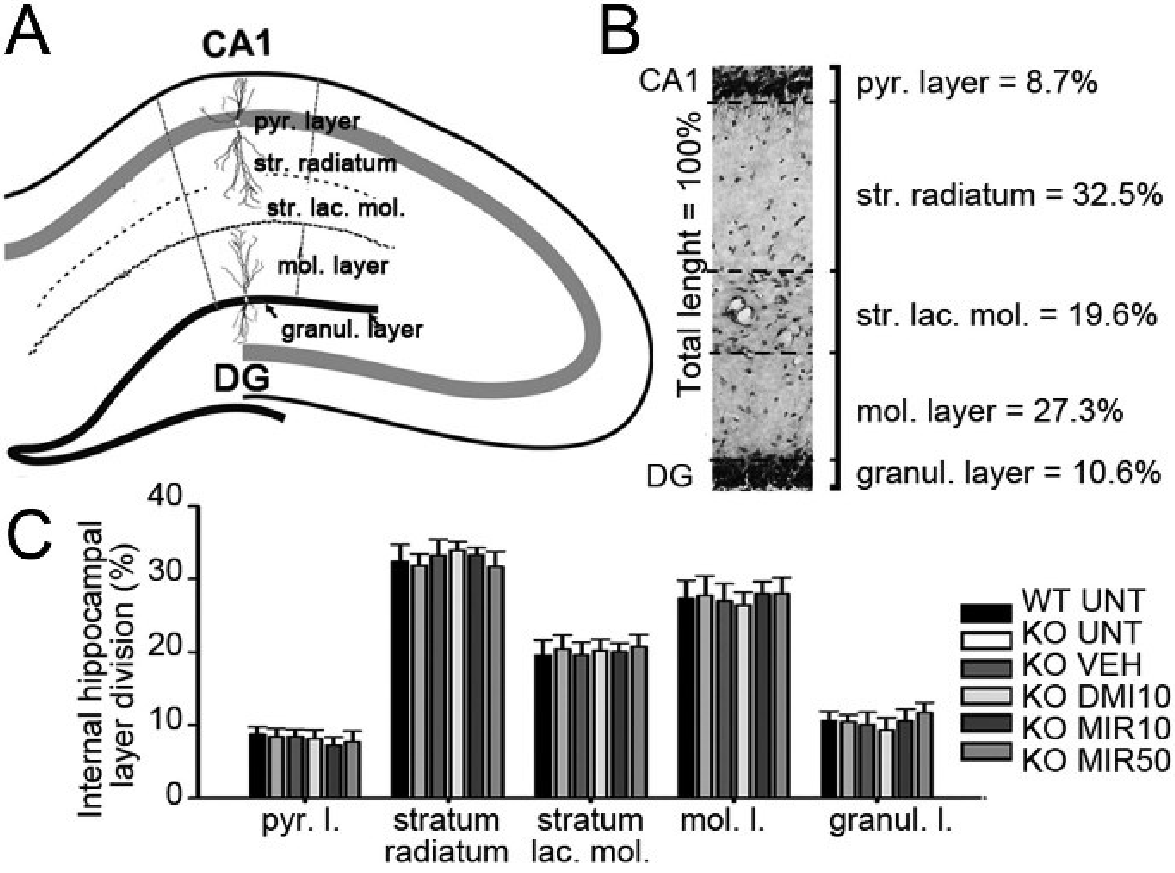

	WT UNT		KO UNT		KO VEH		KO DMI10		KO MIR10		KO MIR50	
%	mean	se	mean	se	mean	se	mean	se	mean	se	mean	se
pyr. l.	8.7	1.08	8.38	1.11	8.41	0.98	8.1	1.2	7.21	1.09	7.71	1.44
strat rad.	32.5	2.27	31.87	1.58	33.26	2.16	34.0	1.2	33.33	1.01	31.69	2.08
s.lac.mol.	19.6	2.05	20.41	1.92	19.59	1.72	20.2	1.5	20.08	1.15	20.76	1.65
mol. l.	27.3	2.50	27.77	2.64	27.01	2.36	26.5	1.8	27.98	1.68	28.00	2.21
granul.l.	10.6	1.25	10.42	0.93	10.06	1.67	9.3	1.7	10.55	1.60	11.68	1.35

Hippocampal structure is not affected by the loss of MeCP2 or Mirtazapine treatment. **(a-b)** A representation of hippocampal structure and layers (*pyramidal layer, stratum radiatum, stratum lacunosum moleculare, molecular layer, granular layer*). **(c)** The proportion of each hippocampal layer based on the total thickness (=100%) in WT and KO untreated (UNT) mice and in KO mice treated with vehicle (vehic), Desipramine 10 mg/Kg (DMI10) and Mirtazapine 10-50 mg/Kg (MIR10-50) (n=3-5). Values are represented as percentage ± SEM (*One way ANOVA*).

Suppl. Figure 2.

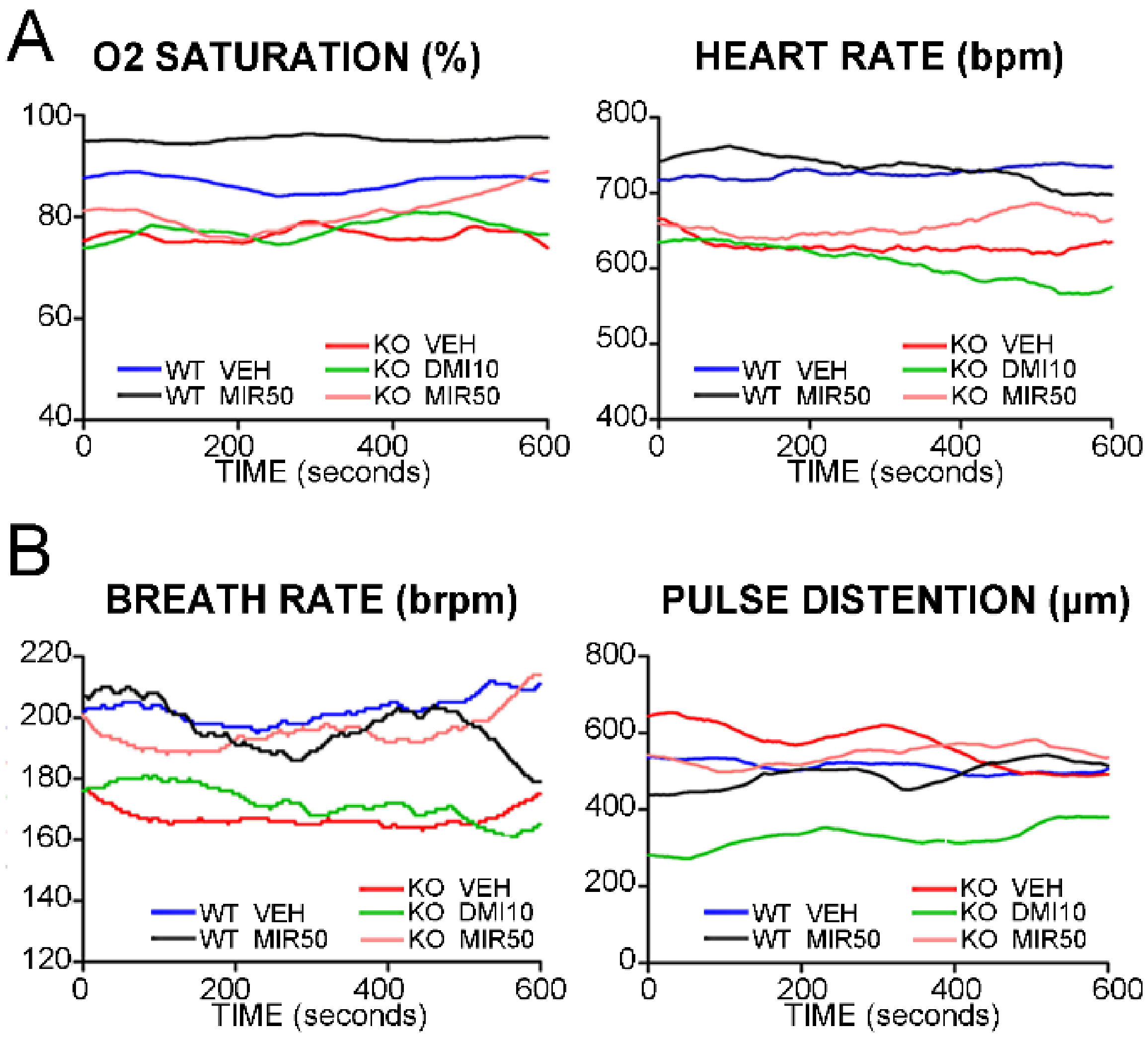

Data profile of the data recording for 13 minutes continuously. WT VEH = line blue; WT MIR50 = black; KO VEH = red; KO DMI10 = green; KO MIR50 = pink. Oxygen saturation (percentage); Heart rate in beat per minute; Breath rate in breath per minute; Pulse distention in µm.
